# Severe vitamin D deficiency is associated with frequent exacerbations and hospitalization in COPD patients

**DOI:** 10.1186/s12931-014-0131-0

**Published:** 2014-12-13

**Authors:** Andrei Malinovschi, Monica Masoero, Michela Bellocchia, Antonio Ciuffreda, Paolo Solidoro, Alessio Mattei, Lorena Mercante, Enrico Heffler, Giovanni Rolla, Caterina Bucca

**Affiliations:** Department of Medical Sciences, University of Turin, Via Lamarmora 41, 10128 Turin, Italy; Department of Medical Sciences, Clinical Physiology, Uppsala University, Uppsala, Sweden; Cardiovascular and Thoracic Department, Città della Salute, Turin, Italy

**Keywords:** Vitamin D, COPD, COPD exacerbation, Hospitalization

## Abstract

**Background:**

Acute exacerbations of COPD (AECOPD) are common and strongly influence disease severity and relative healthcare costs. Vitamin D deficiency is frequent among COPD patients and its contributory role in disease exacerbations is widely debated. Our aim was to assess the relationship of serum vitamin D levels with COPD severity and AECOPD.

**Methods:**

Serum vitamin D (25-hydroxyvitamin D) levels were measured in 97 COPD patients and related to lung function, comorbidities, FEV1 decline, AECOPD and hospital admission during the previous year.

**Results:**

Most patients (96%) had vitamin D deficiency, which was severe in 35 (36%). No significant relationship was found between vitamin D and FEV1 or annual FEV1 decline. No difference between patients with and without severe vitamin D deficiency was found in age, gender, BMI, smoking history, lung function, and comorbidities, apart from osteoporosis (60.9% in severe deficiency vs 22.7%, p = 0.001). In multiple logistic regression models, severe deficiency was independently associated with AECOPD [adjusted odds ratios (aOR) of 30.5 (95% CI 5.55, 168), p < 0.001] and hospitalization [aOR 3.83 (95% CI 1.29, 11.4), p = 0.02]. The odds ratio of being a frequent exacerbator if having severe vitamin D deficiency was 18.1 (95% CI 4.98, 65.8) (p < 0.001), while that of hospitalization was 4.57 (95% CI 1.83, 11.4) (p = 0.001).

**Conclusions:**

In COPD patients severe vitamin D deficiency was related to more frequent disease exacerbations and hospitalization during the year previous to the measurement of vitamin D. This association was independent of patients’ characteristics and comorbidities.

**Electronic supplementary material:**

The online version of this article (doi:10.1186/s12931-014-0131-0) contains supplementary material, which is available to authorized users.

## Background

The prevalence of COPD is increasing and this has a heavy influence on healthcare costs [1], particularly because of the frequent disease exacerbations (AECOPD) and hospital admissions. The disease control is far from being reached and dietary factors are acknowledged as one of the several factors contributing to COPD [2].

Vitamin D deficiency was highly prevalent in a general US population in the third National Health and Examination Survey [3]. According to Janssens *et al.* [4], vitamin D deficiency occurs in over 60% of patients with severe COPD, and is quantitatively related to disease severity. Epidemiological studies indicate that decreased vitamin D is associated with increased frequency of respiratory infections not only in COPD patients, but also in healthy people [4,5]. This may be due to the involvement of vitamin D in both innate and adaptive immunity regulation [6,7]. A general population study by Skaaby *et al.* [8], demonstrated a significant inverse association between vitamin D status and death caused by diseases of the respiratory and digestive system and by endocrine, nutritional and metabolic diseases. Nevertheless, the role of vitamin D in AECOPD is still debated. In a secondary analysis of a study performed in exacerbation-prone COPD patients [9], no association between baseline vitamin D levels and subsequent risk of acute exacerbations was found; negative results have also been reported in a primary care setting [10]. A single center randomized trial on 182 COPD patients [11] demonstrated that vitamin D supplements were able to reduce COPD exacerbations only in the 30 subjects with severe deficiency. These findings leave open the question of the role of vitamin D deficiency and the benefit of its correction in COPD.

We performed a retrospective observational cohort study in COPD patients not taking vitamin D supplements, to evaluate if low vitamin D levels is associated with severe airway obstruction, annual FEV1 decline, disease exacerbations and hospital admission over the course of one year.

## Materials and methods

Patients were selected among 229 consecutive COPD patients, with any range of severity according to the GOLD classification [2], who presented for a scheduled visit at our Respiratory Clinic during the period October 2011-March 2012. Inclusion criteria were age over 40 years, a post-bronchodilator ratio of forced expiratory volume in 1 second (FEV_1_) to vital capacity (VC) <0.7, and at least one year follow-up in our clinic. Exclusion criteria were AECOPD in the last month (n = 19), current treatment with vitamins and dietary supplements (n = 46) and lack of availability for data on lung function/exacerbations/hospitalization the year previous to the inclusion in the study (n = 67), resulting therefore in 97 patients to be included in the study.

The study was approved by the Institutional Review Board (CEI N. 414) and written informed consent was obtained from each patient.

At the enrolment visit, patients underwent clinical examination, recording of symptoms, smoking habits, medication use, lung function tests, venous blood sampling for nutritional assessments. Subjects were classified as current, former- and never-smokers, according to self-reported smoking history. Body mass index (BMI) was calculated as weight divided by height squared (kg/m^2^).

Comorbidities were recorded on the basis of prior diagnosis and current treatment for: systemic arterial hypertension, diabetes, dyslipidemia, anxiety and/or depression, chronic kidney disease, cerebrovascular disease, osteoporosis, obstructive sleep apnoea (OSA), any type of active malignant tumor. The diagnosis of pulmonary hypertension, heart disease and chronic heart failure had to be supported by symptoms and clinical and echocardiographic examination [12].

The medical records of the patients were collected and reviewed retrospectively, to gain information relative to the previous year:

- annual FEV1 decline: difference between actual FEV1 and FEV1 recorded 12 months before.
- annual number of AECOPD, defined on the basis of unscheduled visits in our clinic for acute worsening of respiratory symptoms, causing changes or increases in medications, use of antibiotics or oral steroids, and/or requiring hospitalization [13,14]. Patients with two or more AECOPDs were defined as frequent exacerbators [15].
- hospital admission for AECOPD in the last year

Lung function tests were measured using the Baires System (Biomedin, Padua, Italy). The values of VC, FEV1, FEV1/VC ratio, and maximal midexpiratory flow-rate (MEF_50_) were computed. VC, FEV1 and MEF_50_ were expressed as% of the predicted value [16]. Annual FEV_1_ decline was calculated as percent of the pre-bronchodilator starting value.

Nutritional assessment consisted of: serum levels of ferritin, folic acid and vitamin B12, measured using the chemiluminescent micro-particle immunoassay (Architect System, Abbott diagnostic division, Longford, Ireland), vitamin D (25-hydroxyvitamin D) levels, measured by the RIA method (25OH Vitamin D total-Ria-CT Kit, DIAsource ImmunoAssay S.A., Louvain, Belgium). Vitamin D levels were regarded as normal (≥30 ng/ml), mild-moderate deficiency ( ≥10, but <30 ng/ml), severe deficiency (<10 ng/ml) [17].

### Statistical analysis

All statistics were performed with STATA/IC 12.1 (StataCorp LP, College station, TX, USA) with the exception of calculation of confidence intervals for sensitivity and specificity at different cut-off levels that was performed with GraphPad Prism v 6.0 (GraphPad Software, San Diego, CA, USA).

Pearson’s chi-squared test was used to compare the prevalence of categorical variables between groups (2 or more groups). t-test or Mann-Whitney-test was used to compare differences in the levels of continuous variables between 2 groups (e.g. males vs females or subjects with and without severe vitamin D deficiency).

Linear regression analyses were performed to analyze the association between (log-transformed) vitamin D levels and lung function indices. These associations were tested in multiple linear regression models after adjustments for potential confounders.

Logistic regression analysis models were used to calculate odds ratios (simple models) or adjusted odds ratios (aOR) for severe vitamin D deficiency in relation to being a frequent exacerbator or being hospitalized for COPD. MEF_50_ was the only lung function parameter included in the models as it had the strongest relationship with vitamin D levels in univariate models. Interaction of severe vitamin D deficiency with gender on the association with being a frequent exacerbator or hospitalized were also tested in the multiple logistic regressions. Receiver-operator characteristic (ROC)-analyses were performed to identify the value of vitamin D with the best combination of specificity and sensitivity in predicting frequent exacerbations and hospitalization for COPD. Several cut-offs are presented: for high sensitivity, high specificity and optimal cut-off, according to the Youden index [18].

Two multiple regression models were created: one including only patient characteristics, the other including also comorbidities and nutritional data. For all the results, a p-value <0.05 was considered to be statistically significant.

## Results

### Patient characteristics

General characteristics of the study patients and by sex, are given in Table 1; comorbidities and nutritional data are reported in Table 2. The study population was gender-balanced, the majority of subjects (62%) were current smokers. More than half of the patients (57%) were in moderate GOLD class, 55% were frequent exacerbators and 52% had been hospitalized at least once in the previous year. The most frequent comorbidities were hypertension and heart disease (HD), found in almost two thirds of the subjects. Vitamin D deficiency (<30 ng/mL) was found in all but four patients and was severe in 36% of them.

**Table 1:** **Characteristics of the overall patients and by gender**

**Variable**	**All patients**	**Men**	**Women**
	**(n = 97)**	**(n = 49)**	**(n = 48)**
Age median (range)	67.5 +/- 10.5	69.1 +/- 10.5	65.8 +/- 10.4
BMI (mean +/- SD)	25.5 ± 5.20	26.3 ± 4.4	24.7 ± 5.8
Smoking habits			
• Current smokers n (%)	60 (61.9%)	27 (55.1)	33 (68.8)
• Former smokers n (%)	22 (22.7%)	18 (36.7)	4 (8.3)*
• Never smokers n (%)	15 (15.6%)	4 (8.2)	11 (22.9)*
GOLD class n (%)			
• 1	18 (18.6)	9 (18.4)	9 (18.8)
• 2	55 (56.7)	27 (55.1)	28 (58.3)
• 3	20 (20.6)	12 (24.5)	8 (16.7)
• 4	4 (4.1)	1 (2.0)	3 (6.3)
Medication for COPD n (%)			
• None	6 (7.4)	1 (2.4)	5 (12.5)
• Long Acting Muscarinic Antagonist (LAMA)	53 (55)	28 (57)	25 (52)
• Long Acting Beta Adrenergic (LABA)	64 (66)	35 (71)	29 (60)
• Inhaled Corticosteroids (ICS)	61 (63)	35 (71)	26 (54)
AECOPD/year, median (IQR)	2 (1-2)	2 (1-2)	2 (1-2.5)
Frequent exacerbators n (%)	55 (56.7)	25 (51.0)	30 (62.5)
Hospitalized for AECOPD in the last year, n (%)	50 (51.6)	25 (51.0)	25 (52.0)
FEV_1_%pred, mean ± SD	62.3 ± 18.0	62.4 ± 17.3	62.2 ± 18.9
VC%pred, mean ± SD	83.1 ± 18.1	82.0 ± 18.3	84.1 ± 18.0
FEV_1_/VC%, mean ± SD	53.8 ± 11.4	54.0 ± 11.5	53.6 ± 11.4
MEF_50_% pred, mean ± SD	28.8 ± 21.4	28.8 ± 20.6	28.8 ± 22.4

*Significant difference (p < 005) between men and women.

**Table 2:** **Comorbidities and nutritional status in the overall patients and by gender**

**Variable**	**All patients**	**Men**	**Women**
	**(n = 97)**	**(n = 49)**	**(n = 48)**
Hypertension n (%)	59 (62.1)	25 (51)	34 (70.8)*
Heart disease (any*) n (%)	64 (67.4)	29 (59.2)	35 (72.9)
• Hypertensive n (%)	26 (27.4)	13 (26.5)	13 (27.1)
• Ischemic n (%)	19 (20.0)	12 (24.5)	7 (14.6)
• Heart failure n (%)	48 (50.5)	24 (49.0)	24 (50.0)
• Valvular n (%)	33 (34.7)	12 (24.5)	21 (43.8)*
• Atrial fibrillation n (%)	22 (23.2)	12 (24.5)	10 (20.8)
Pulmonary hypertension n (%)	23 (24.2)	10 (20.4)	13 (27.1)
Dyslipidemia n (%)	33 (34.7)	15 (30.6)	18 (37.5)
Thyroid disease n (%)	20 (20.6)	5 (10.2)	15 (31.3)*
Diabetes n (%)	15 (15.8)	8 (16.3)	7 (14.6)
Cerebrovascular disease n (%)	11 (11.3)	8 (16.3)	3 (6.3)
Renal failure n (%)	17 (20.2)	10 (20.4)	7 (14.6)
Osteoporosis n (%)	23 (25.8)	8 (16.3)	15 (31.3)
Cancer n (%)	17 (17.7)	11 (22.9)	6 (12.5)
Depression/anxiety n (%)	28 (29.5)	11 (22.4)	17 (35.4)
GERD n (%)	15 (16.7)	5 (10.2)	10 (20.8)
Obstructive sleep apnea n (%)	11 (12.4)	5 (10.2)	6 (12.5)
**Nutrition parameters**			
Vitamin D levels (ng/ml) (GM (95%CI)]	12.0 (10.6 - 13.5)	15.5 (12.7-18.4)	12.7 (10.9-14.6)
Mild-moderate deficiency n (%)	58 (57.9%)	31 (63.3)	27 (56.3)
Severe deficiency n (%)	35 (36.1%)	14 (28.6)	21 (43.8)*
Ferritin deficiency (<25 mg/dl) n (%)^#^	10 (11.1%)	4 (8.5)	6 (14.0)
Vitamin B12 deficiency (<200 pg/ml) n (%)^#^	19 (20.2%)	11 (22.9)	8 (17.4)
Folic acid deficiency (<4.5 ng7ml) n (%)^#^	33 (35.9%)	16 (34.0)	17 (37.8)

*Data on comorbidities available in 84 to 97 subjects, depending on comorbidity.

^#^Ferritin levels measured in 90 subjects, Vitamin B12 in 94 subjects, Folic acid in 92 subjects.

Women, as compared to men, had higher prevalence of never smokers, severe vitamin D deficiency, thyroid disease, hypertension, valvular heart disease, and a trend towards a larger number of AECOPD (p = 0.07).

The comparison between patients with (n = 35) and without severe (n = 62) vitamin D deficiency showed no significant difference regarding age, BMI, smoking history and comorbidities (all p-values over 0.10) apart from osteoporosis, significantly more frequent in severe deficiency (60.9% vs 22.7%, p = 0.001). Likewise, no significant difference was found in FEV_1_% predicted (p = 0.27), GOLD class (p = 0.16), and VC% predicted (p = 0.49); however, subjects with severe deficiency had significantly lower MEF_50_ (22.3 +/- 15.8% vs 32.5 +/- 23.3%, p = 0.02) and a trend toward lower FEV_1_/VC ratio (51.2% +/- 11.5% vs. 55.3% +/ 11.2, p = 0.09).

**Figure 1:**
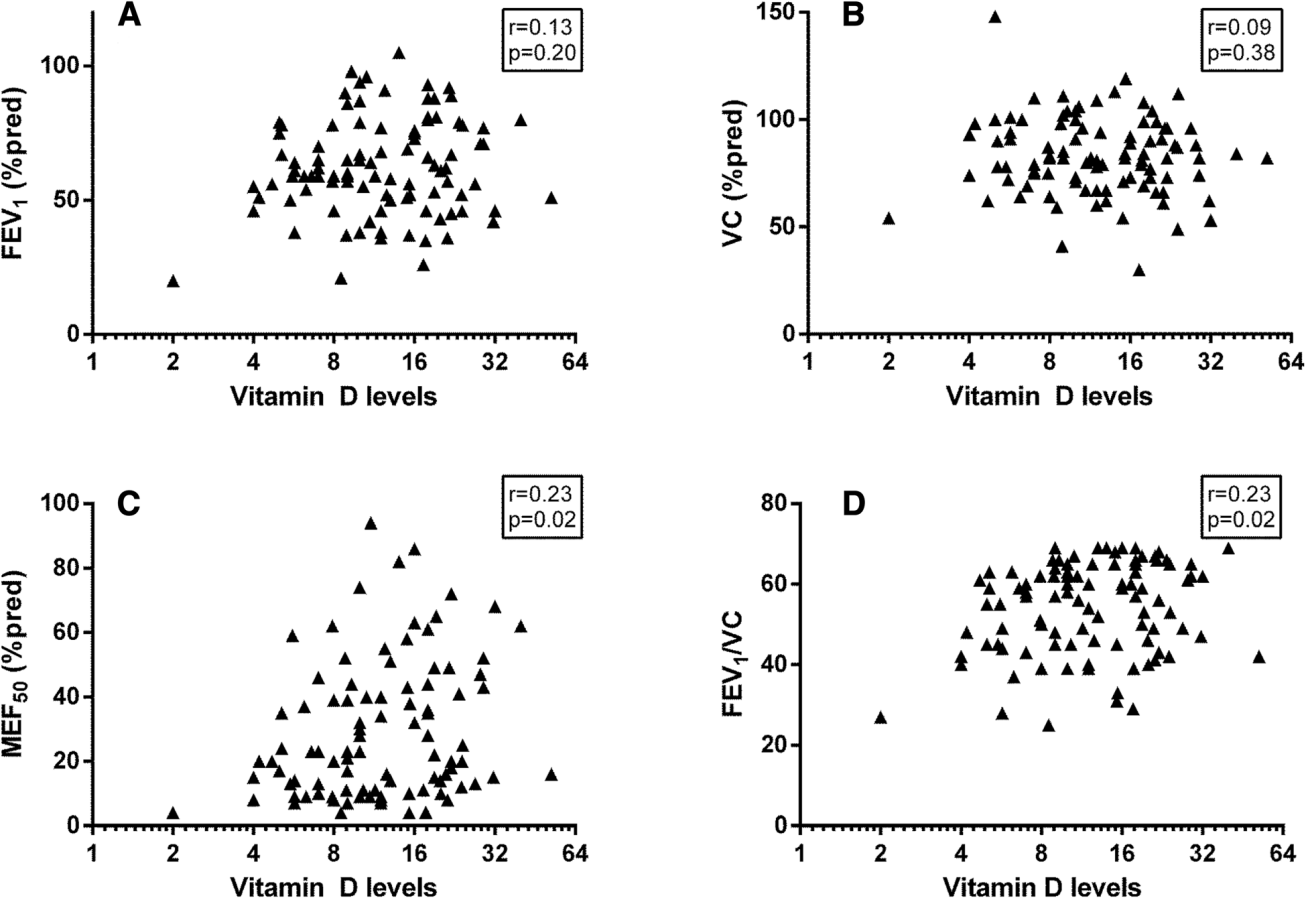
**Lung function parameters: FEV**_**1**_ **(%predicted) (A), VC (% predicted) (B), MEF**_**50**_ **(% predicted) (C), FEV**_**1**_ **/VC (×100) (D) in relation to vitamin D levels (log-scale).**

As shown in Figure 1, vitamin D levels were not related to FEV_1_% predicted (p = 0.20) (Figure 1A) and VC% predicted (p = 0.38) (Figure 1B), but were significantly negatively related to MEF_50_ levels (p = 0.02) (Figure 1C) and FEV_1_/VC ratio (p = 0.02) (Figure 1D). However, after adjusting for gender, age, BMI and smoking habits, all the relationships presented in Figure 1, all relations were not significant (p > 0.05), and remained so also after further adjustment for comorbidities (p > 0.05).

No significant relation could be found between vitamin D levels and fall in FEV_1_ during the year preceding vitamin D measurements, expressed either as absolute levels (p = 0.20) or as percent fall (p = 0.09), even after adjusting for gender, age, BMI and current smoking (p = 0.35 for absolute value and p = 0.26 for FEV_1_% fall). Levels of FEV_1_ fall were similar in subjects with and without severe vitamin D deficiency (p = 0.51 for absolute and p = 0.36 for percent fall).

**Figure 2:**
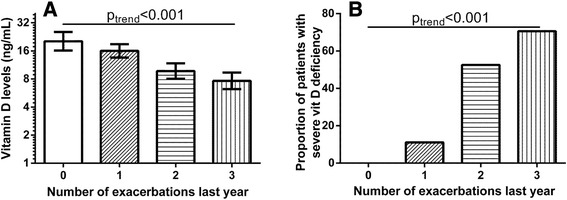
**Levels of vitamin D (log-scale) in COPD subjects divided according to the number of exacerbations the year previous to the measurements of vitamin D levels (A) and the proportion of subjects with severe vitamin D deficiency (<10 ng/mL) in COPD subjects divided according to the number of exacerbations the year previous to the measurements of vitamin D levels (B).**

An analysis of vitamin D levels stratified by number of exacerbations in the year preceding the study entry showed that the higher the number of AECOPD the lower the vitamin D level (p < 0.001) (Figure 2A). This relation persisted after adjusting for gender, age, smoking habits, and lung function (MEF_50_) (p < 0.001), and after further adjusting for other nutritional deficiencies and comorbidities (p < 0.001). Similarly, the proportion of subjects with severe vitamin D deficiency increased with the number of AECOPD (p < 0.001) (Figure 2B).

The prevalence of severe vitamin D deficiency was larger among frequent than among non-frequent exacerbators (58.2% versus 7.1%, p < 0.001). The odds ratio of being a frequent exacerbator if having severe vitamin D deficiency was 18.1 (95% CI 4.98, 65.8) (p < 0.001). Severe vitamin D deficiency was independent of frequent exacerbations [adjusted OR = 30.5 (95% CI 5.55, 168), p < 0.001] in a multiple logistic regression model after adjusting for gender, age, BMI, smoking, lung function (MEF_50_), even after further adjusting for nutritional deficiencies [adjusted OR = 34.9 (95% CI 4.89, 249), p < 0.001]. No interaction between severe vitamin D deficiency and gender was found in this model (p = 0.84). The relation between severe vitamin D deficiency and frequent exacerbations was consistent after further adjusting for all the comorbidities (p = 0.03).

**Figure 3:**
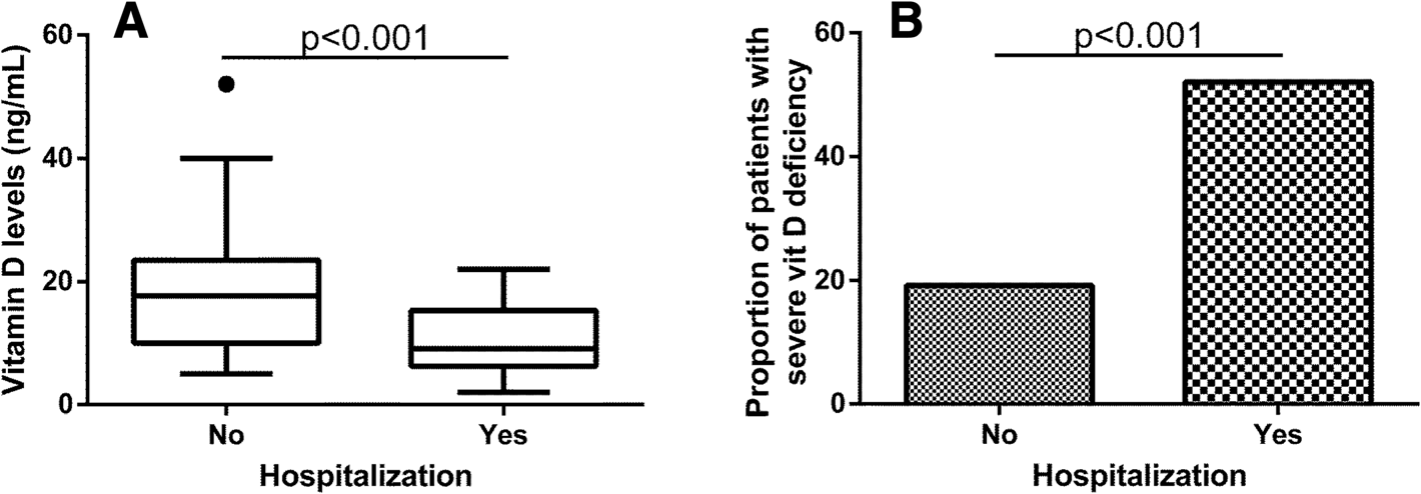
**Levels of vitamin D (A) or prevalence of severe vitamin D deficiency (B) in COPD subjects divided according to being hospitalized the year previous to the measurements of vitamin D levels.**

A total of 50 subjects (51.5%) were hospitalized for AECOPD at least once during the year preceding vitamin D measurement. These subjects had significantly lower vitamin D levels (p < 0.001) (Figure 3A). and higher prevalence of severe vitamin D deficiency (p = 0.001) (Figure 3B). Lower levels of vitamin D were associated with hospitalization (p < 0.001) after adjusting for gender, age, BMI, smoking, lung function (MEF_50_) (p = 0.002), and the association persisted after further adjusting for comorbidities (p = 0.001). No differences in vitamin D levels (p = 0.74) or prevalence of severe vitamin D deficiency (p = 0.85) were found between subjects hospitalized once (n = 34) or twice (n = 16) during the preceding year.

ROC curve for vitamin D levels in relation to frequent exacerbations yielded an area under the curve (AUC) of 0.83 (95% CI 0.75, 0.91) (Additional file 1: Figure S1). The optimal cut-off (according to Youden index) to identify frequent exacerbators was 9.15 ng/mL (Additional file 2: Table S1). All the subjects (n = 27) with vitamin D levels below 8.65 ng/mL were frequent exacerbators, while 9 of 10 subjects with two or more AECOPD in the last year had vitamin D levels below 19.15 ng/mL (Additional file 2: Table S1). Similarly, low vitamin D was associated with any AECOPD [AUC = 0.81 (95% CI 0.71, 0.91)] and all subjects with vitamin D levels below 11.7 ng/mL had at least one AECOPD during the year preceding the study (Additional file 2: Table S2).

The odds ratio of being hospitalized if having severe vitamin D deficiency was 4.57 (95% CI 1.83, 11.4) (p = 0.001). Severe vitamin D deficiency wasindependently related with hospitalizations [aOR 3.83 (95% CI 1.29, 11.4)] after adjusting for gender, age, BMI, smoking and lung function (MEF_50_) (p = 0.01). This effect was consistent after further adjustment for nutritional deficiencies [adjusted OR 8.45 (95% CI 1.82, 39.2), p = 0.006] and no interaction between severe vitamin Ddeficiency and gender was found in this model (p = 0.50). The relationship between severe vitamin D deficiency and hospitalization was consistent even after adjusting for comorbidities (p = 0.02).

ROC curve for vitamin D levels in relation to hospitalization yielded an AUC of 0.75 (0.65, 0.84) (Additional file 3: Figure S2). The optimal cut-off value to identify subjects hospitalized for AECOPD was 12.2 ng/ml, and 9 out of 10 subjects who had been hospitalized during the last year had vitamin D levels below 19.15 ng/mL (Additional file 2: Table S3).

## Discussion

The results of the study demonstrate that most COPD patients had vitamin D deficiency. Severe deficiency was associated with frequent exacerbations, documented in our clinic, and hospitalization during the previous year. These findings were consistent even after adjustments for comorbidities and other nutrient deficiencies. Vitamin D levels were not related to either lung function at the study enrolment or lung function decline in the preceding year.

Vitamin D deficiency represented the major nutritional disorder in our study population, being mild-moderate in about 60% and severe in as high as 36% of the patients. A high prevalence of vitamin D deficiency in COPD patients has been previously reported [4,9,11,19], but never as high as in our patients. This may depend on several reasons. First, vitamin D was deliberately assessed in the winter season, when levels are supposed to be lowest [20], especially to the latitude of our city [21,22]. Anyway, it is presumable that our subjects were deficient throughout the year, as in Italy, at variance with other countries [23], there is no food fortification with vitamin D. It has been estimated that a summer vitamin D level around 40 ng/mL is needed to achieve a 20 ng/mL concentration the following winter [24] and similar variations appear to exist in COPD patients, with approximately 35% lower values in winter compared to summer [25]. The lowest winter vitamin D values may contribute to the marked impact of winter season on both frequency and outcomes of COPD exacerbations [26,27].

These findings are in agreement with prior observations that low vitamin D levels are associated with increased frequency of respiratory infections in both COPD patients and healthy adults [4,5,28]. However, the role of vitamin D deficiency in AECOPD is still debated. A recent study on exacerbation-prone COPD patients found no association between baseline vitamin D levels and subsequent risk of AECOPD [9]. However, according to Heulens *et al.* [29], this negative finding might depend on the fact that some of the patients with worse clinical conditions were taking vitamin D supplements. Of course, once supplemented, vitamin D levels no longer reflect the underlying COPD severity. Actually, excluding those taking supplements from the analysis, Heulens *et al.* [29] could demonstrate that patients with vitamin D levels below 10 ng/ml had the shortest time to first exacerbation and experienced the highest number of AECOPD. Moreover, pooling together patients with vitamin D levels ranging from 10 to 30 ng/ml, they found a dose–response relationship for exacerbations number very similar to that found in our study. Another recent study by Puhan *et al.* [10] reported no relation of severe vitamin D deficiency with exacerbations and no effect of vitamin D supplements on AECOPD. Such negative result may depend on several reasons. First, vitamin D deficiency was less severe than that observed in our study patients (15.5 versus 12 ng/dl). Second, although patients taking vitamin D supplements had been excluded at enrolment, it was not specified if patients were supplemented during the biannual follow-up. Furthermore, different vitamin D assays were used in these studies and this could, at least in theory, yield different results and therefore vitamin D levels in absolute values might be more difficult to compare between studies. A recent study on 12,041 individuals from the Danish general population by Skaaby *et al.* [30], found that vitamin D status was significantly inversely associated with COPD but had no influence on COPD incidence. This finding suggests that vitamin D deficiency is a consequence rather than a cause of COPD. The effect of vitamin D supplementation in COPD is still debated. High dose vitamin D supplementation has been found to to decrease AECOPD number, but only in patients with severe deficiency [11] and to improve inspiratory muscle strength and maximal oxygen uptake [31].

At variance with other findings [19,32], in our patients vitamin D levels were not related to FEV_1_ and to the GOLD-COPD class, which is based on FEV_1_ value, neither were they predictive of FEV_1_ decline. This could simply be due to the fact that the size of such effect is so small that a larger population would have been needed to demonstrate it. Actually, both the studies of Persson *et al.* [19] and Berg *et al.* [32] had populations about five times larger than ours, and patients were well distributed among the GOLD classes, while most of our patients were in GOLD class II. Interestingly, in univariate analysis the levels of vitamin D were significantly related to both FEV_1_/VC and MEF_50_, but these correlations disappeared after adjustments for potential confounders.

The major weakness of the present study was its retrospective design, as the vitamin D levels were measured at the end of the observation period, where events (exacerbations) and lung function decline were recorded. Therefore a potential reverse causation effect, with exacerbations lowering levels of vitamin D, cannot be excluded even if no such relation has been described in the literature [25]. Furthermore, a study in independently living community-dwelling subjects of similar age, with different degrees of vitamin D deficiency, reported steady levels over a one year period [33] and therefore it might be speculated that the levels of vitamin D measured in the present study could be similar to the previous year values. However, in our opinion, a retrospective study, as compared to a prospective one, avoids the criticism of not treating patients with severe deficiency.

## Conclusions

The results of the present study demonstrate that vitamin D deficiency is very common in COPD patients, and indicate that severe deficiency is associated with higher probability having frequent exacerbations and hospitalization and this association is independent of patient characteristics and comorbidities. The prevention of exacerbations is a major treatment goal of COPD and the benefit of vitamin D supplementation, particularly during the winter season, is an intervention that warrants further assessment. However randomized controlled trials are needed to clarify whether the observed association in the present study is causal and is really attributable to vitamin D deficiency.

## Additional files

Additional file 1: Figure S1. Receiver operating characteristic (ROC) curve for the value of vitamin D levels to identify subjects who were frequent exacerbators the year previous to the measurements of vitamin D levels (≥2/year). Additional file 2: Table S1. Sensitivity and specificity (95% CI) for different cut-offs of vitamin D levels to identify frequent exacerbators. **Table S2.** Sensitivity and specificity (95% CI) for different cut-offs of vitamin D levels to identify subjects who had any exacerbation the year previous to the vitamin D measurements. **Table S3.** Sensitivity and specificity (95% CI) for different cut-offs of vitamin D levels to identify subjects hospitalized the year previous to the vitamin D measurements. Additional file 3: Figure S2. Receiver operating characteristic (ROC) curve for the value of vitamin D levels to identify subjects who were hospitalized during the year preceding vitamin D measurement.

## Abbreviations

AECOPD: Acute COPD exacerbations
AUC: Area under the curve
aOR: Adjusted odds ratio
BMI: Body mass index
COPD: Chronic obstructive pulmonary disease
FEV_1_: Forced expiratory volume in 1 second
GOLD: Global initiative for obstructive lung disease
ICS: Inhaled corticosteroids
LABA: Long acting beta agonist
LAMA: Long acting muscarinic antagonist
VC: Vital capacity
