# Supplementary figures and images for: Severe vitamin D deficiency is associated with frequent exacerbations and hospitalization in COPD patients
Source: Respir Res. 2014 Dec 13;15(1):131. doi: 10.1186/s12931-014-0131-0 (PMC4269938; doi:10.1186/s12931-014-0131-0)

## Slide 1
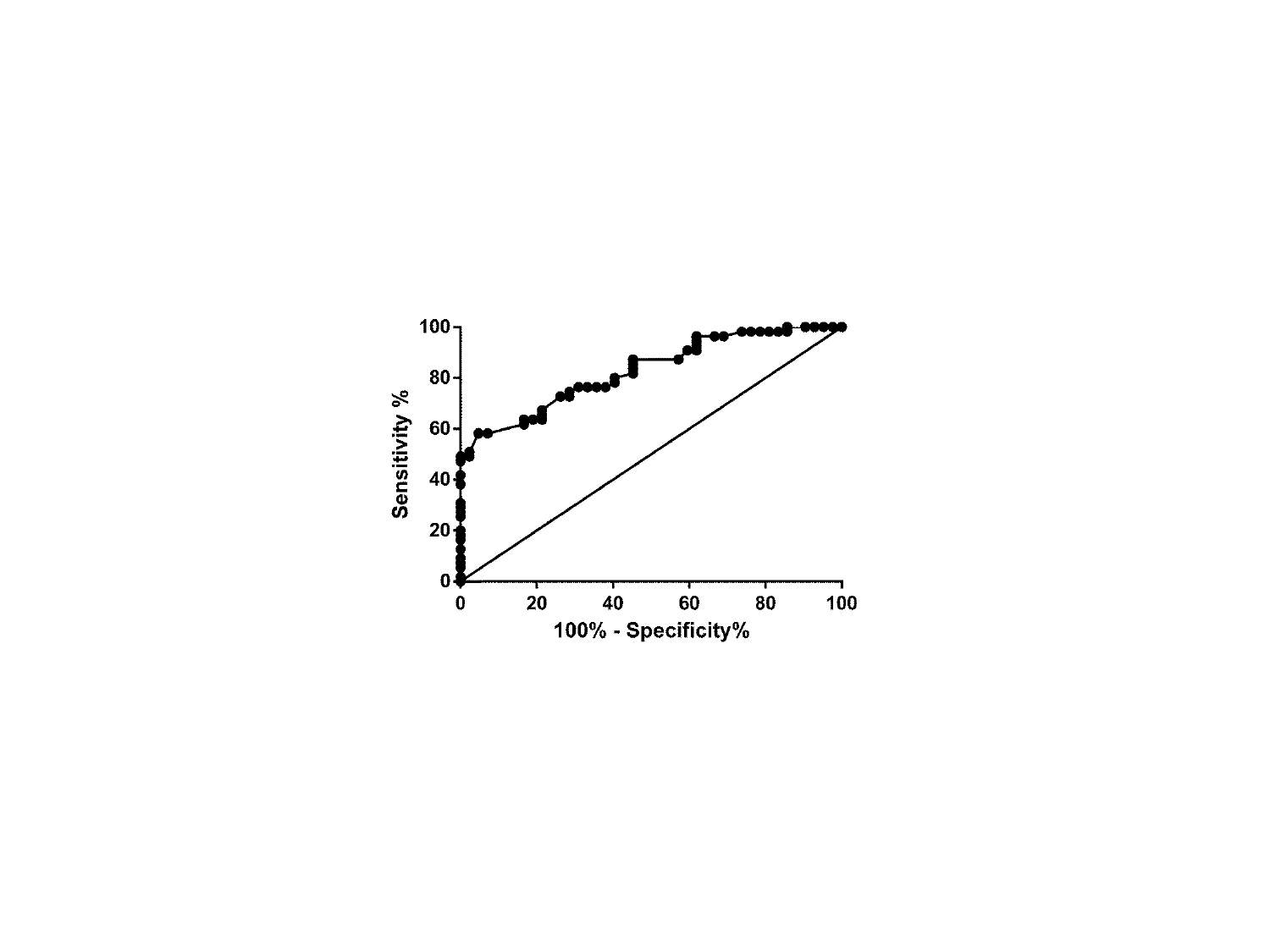

Supplement: Additional file 1: Figure S1. — Receiver operating characteristic (ROC) curve for the value of vitamin D levels to identify subjects who were frequent exacerbators the year previous to the measurements of vitamin D levels (≥2/year). [file 12931_2014_131_MOESM1_ESM.pptx]

## Slide 1
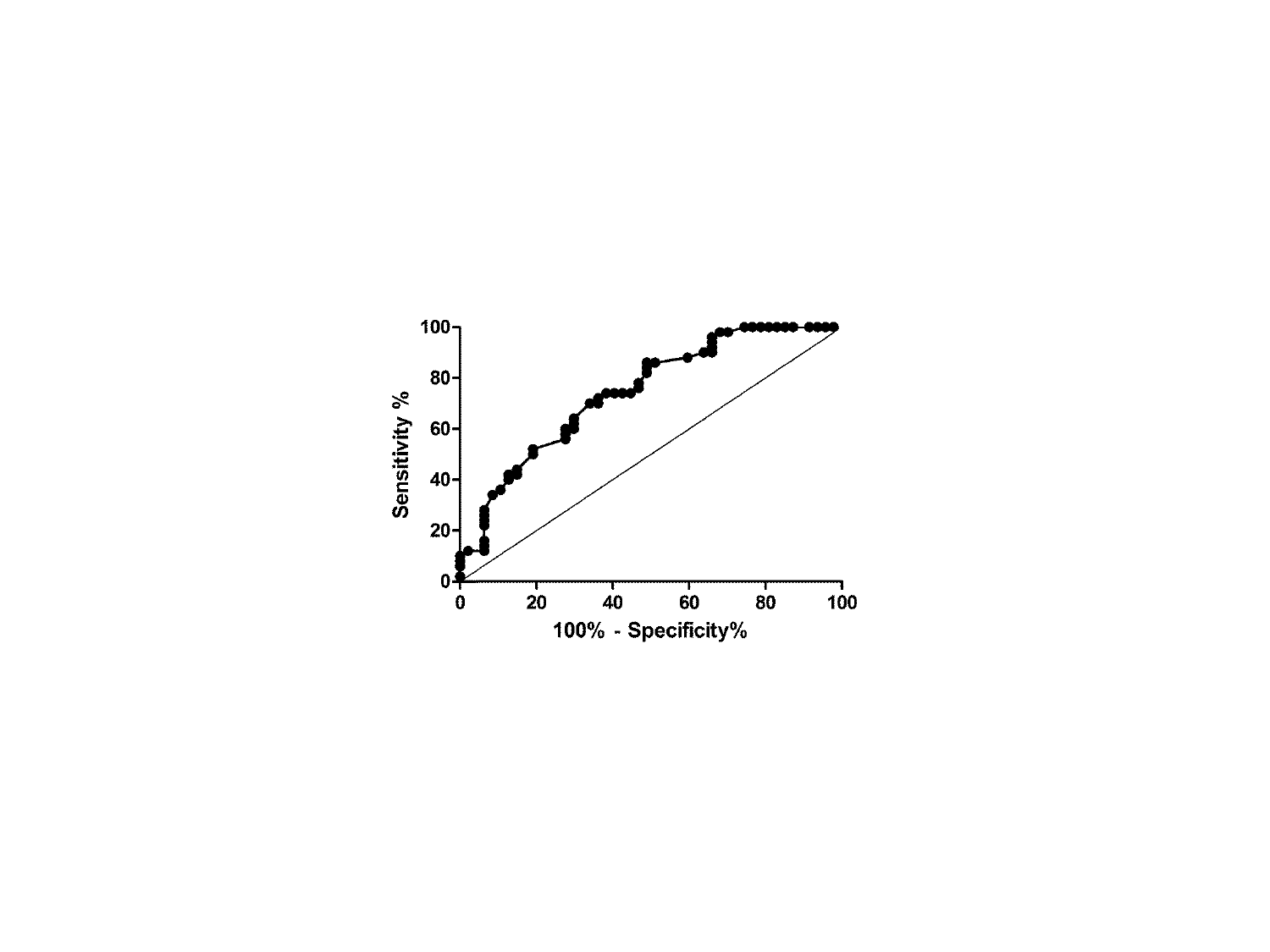

Supplement: Additional file 3: Figure S2. — Receiver operating characteristic (ROC) curve for the value of vitamin D levels to identify subjects who were hospitalized during the year preceding vitamin D measurement. [file 12931_2014_131_MOESM3_ESM.pptx]
