# Supplementary material for: Severe vitamin D deficiency is associated with frequent exacerbations and hospitalization in COPD patients
Source: Respir Res. 2014 Dec 13;15(1):131. doi: 10.1186/s12931-014-0131-0 (PMC4269938; doi:10.1186/s12931-014-0131-0)
Supplement: Additional file 2: Table S1. — Sensitivity and specificity (95% CI) for different cut-offs of vitamin D levels to identify frequent exacerbators. Table S2. Sensitivity and specificity (95% CI) for different cut-offs of vitamin D levels to identify subjects who had any exacerbation the year previous to the vitamin D measurements. Table S3. Sensitivity and specificity (95% CI) for different cut-offs of vitamin D levels to identify subjects hospitalized the year previous to the vitamin D measurements. [file 12931_2014_131_MOESM2_ESM.docx]

**Additional file 2: Table S1. Sensitivity and specificity (95%CI) for different cut-offs of vitamin D levels to identify frequent exacerbators.**

|  | Cut-off value | Sensitivity (95%CI) | Specificity (95% CI) |
| --- | --- | --- | --- |
| Optimal cut-off | <9.15 | 58.2% (44.1, 71.4%) | 95.2% (83.8, 99.4%) |
| 100% specificity | <8.65 | 49.1% (35.4, 62.9%) | 100% (91.6, 100%) |
| 90% specificity | <9.65 | 58.2% (44.1, 71.4%) | 92.9% (80.5, 98.5%) |
| 90% sensitivity | <19.15 | 90.9 % (80.1, 97.0%) | 40.5% (25.6, 56.7%) |

**Additional file 2: Table S2. Sensitivity and specificity (95%CI) for different cut-offs of vitamin D levels to identify subjects who had any exacerbation the year previous to the vitamin D measurements.**

|  | Cut-off value | Sensitivity (95%CI) | Specificity (95% CI) |
| --- | --- | --- | --- |
| Optimal cut-off | <11.7 | 56.1 % (44.7, 67.1%) | 100% (78.2, 100%) |
| 100% specificity | <11.7 | 56.1 % (44.7, 67.1%) | 100% (78.2, 100%) |
| 90% specificity | <12.2 | 59.8% (48.3, 70.4%) | 86.7% (59.5, 98.3%) |
| 90% sensitivity | < 22.75 | 91.5 % (83.2, 96.5%) | 33.3% (11.8, 61.6%) |

**Additional file 2: Table S3. Sensitivity and specificity (95%CI) for different cut-offs of vitamin D levels to identify subjects hospitalized the year previous to the vitamin D measurements.**

|  | Cut-off value | Sensitivity (95%CI) | Specificity (95% CI) |
| --- | --- | --- | --- |
| Optimal cut-off | <12.2 | 70 % (55.4, 82.1%) | 66.0% (50.7, 79.1%) |
| 100% specificity | <4.85 | 10 % (3.3, 21.8%) | 100% (92.5, 100%) |
| 90% specificity | <7.45 | 34% (21.2, 48.8%) | 91.5% (79.6, 97.6%) |
| 90% sensitivity | <19.15 | 90.0 % (78.2, 96.7%) | 36.2% (22.7, 51.5%) |
